# Prospective healthcare students' perspectives and attitudes on pharmacy as a chosen career path

**DOI:** 10.1016/j.rcsop.2024.100512

**Published:** 2024-09-25

**Authors:** Shadi Doroudgar, Mahsa Sedaghat, Nabeez Noor, Shervin Gorji

**Affiliations:** Touro University California College of Pharmacy, Vallejo, California, USA

**Keywords:** Pharmacy enrollment, Prospective students, Pharmacy candidates

## Abstract

**Background:**

With the national increase in the number of colleges and schools of pharmacy as well as the decreased pharmacy school applicant pool in recent years, pharmacy school enrollment challenges are at the forefront of issues facing pharmacy programs today. Factors that can influence a candidate's likelihood to pursue pharmacy school are important to evaluate. Recent studies assessing factors that motivate potential pre-health candidates to pursue pharmacy school or other healthcare careers are limited.

**Objective:**

To describe prospective candidates' perspectives and attitudes toward pharmacy as a career pathway and assess factors that motivate or deter them from pursuing pharmacy as a career and from considering a specific pharmacy school.

**Method:**

The study was cross-sectional and used an online data collection form distributed via the Qualtrics platform from October 4, 2022 through October 12, 2022 using the social media platform Reddit. Reddit has hundreds of subcommunities known as subreddits, that allow for exchange of ideas and discussion regarding various topics. This social media platform is commonly used by pre-health students to discuss career options. The survey consisted of 28 items and two domains, demographics and prospective healthcare students' understanding of responsibilities of pharmacists and their overall perspective on pharmacy as a profession. The survey was targeted toward anyone with an intention of pursuing a health science degree or career.

**Result:**

Four hundred participants completed the survey. Participants were mainly Caucasian, male, and with a mean age of 25 years. This study assessed prospective candidates' perspectives and attitudes toward pharmacy as a career pathway, appealing and unappealing characteristics about pursuing a career in the field of pharmacy, and factors that motivate or deter candidates from pursuing pharmacy. While 68 % of the participants had heard that career choices are vast and vary in the field of pharmacy, most still had concerns about the pharmacy field being heavily saturated and lack of job opportunities after graduation. Almost 60 % of responders gained information about pharmacy programs through social media platforms. The North American Pharmacist Licensure Examination pass rate, postgraduate residencies/fellowships, program duration and program reputation were among the top factors in choosing a pharmacy program.

**Conclusion:**

Many survey participants had heard that there are a variety of job opportunities in the field of pharmacy, but the majority had heard that the pharmacy field is heavily saturated and that there are lack of opportunities after graduation. Most responders gained information through social media platforms. For prospective pharmacy school candidates, NAPLEX pass rate, number of graduates matched with PGY1 residencies and fellowships, and duration and reputation of the program seems to be among the most important factors in choosing a pharmacy program. Factors that prospective candidates consider when selecting a pharmacy program and the means they use to research about programs can be important considerations for pharmacy programs recruitment. Awareness of the preferences and baseline knowledge of candidates about the field of pharmacy can aid in developing targeted recruitment strategies and informing decision-making in pharmacy programs and recruitment efforts.

## 1 Introduction

The number of colleges and schools of pharmacy has increased over the past few decades.^[^^1^^]^ The total number of colleges and schools of pharmacy in the United States has increased from 110 for the academic year of 2012–2013 to 146 as of 2023.^[^^2^^,^^3^^]^ In California, the number of pharmacy schools has grown from eight in 2013 to 14 in 2023.^[^^3^^,^^4^^]^ Although the number of pharmacy schools has increased, the number of applicants to colleges of pharmacy has declined. This has led to difficulties with attracting and adequately recruiting qualified candidates to the field of pharmacy.^[^^5^^]^ Despite the increased acceptance rate of pharmacy programs, according to American Association of Colleges of Pharmacy (AACP), the Pharmacy College Application Service (PharmCAS) reports a steady decline in the number of pharmacy applicants over the past decades.^[^^6^^]^ During the 2011–2012 cycle, the acceptance rate in pharmacy programs was 62.1 %. This rate increased to 86.5 % for 2021–2022 cycle.^[^^6^^]^ The number of PharmCAS applicants in 2011–2012 cycle was 17,405. This number decreased to 11,219 applicants in 2021–2022 cycle.^[^^6^^]^ That equates to almost a 36 % decline in pharmacy school applicants over a 10-year span.^[^^6^^]^ Given this dynamic, applicants are aware that they are likely to obtain admission at their school of choice and are applying to far fewer pharmacy programs.^[^^7^^]^

To attract candidates, individual pharmacy programs must compete with other pharmacy programs and other health profession programs.^[^^8^^]^ Outside of pharmacy, other healthcare professions such as physician assistant (PA) have had growth in their number of workers and a dramatic rise of submitted applicants.^[^^9^^,^^10^^]^ For instance, in the United States, the PA profession grew 28.7 % between 2017 and 2021, reaching 158,470 certified PAs at the end of 2021.^[^^10^^]^ This rise in demand of PAs and other mid-level providers has been partly due to compensating for shortages of health-care providers such as primary care providers and nurses. The COVID-19 pandemic has also contributed to the diminishing pharmacy school applicant pool.^[^^11^^]^ This unforeseen event caused further complications for programs that rely on recruitment strategies that involve direct human interaction. Many of the in-person career fairs and face-to-face interactions of recruiters were halted during the COVID-19 pandemic. Additionally, many of the pre-pharmacy organizations' activities at the undergraduate institution level were either paused or shifted online. As such, a greater emphasis was placed on finding information online, via social media platforms, program websites, or virtual recruitment events.

Studies evaluating factors that motivate potential pre-health candidates to pursue pharmacy school or other healthcare careers would benefit faculty and staff at colleges of pharmacy by helping them to learn more about students' overall attitude toward those programs. Research on characteristics that are appealing and differentiate pharmacy school programs would also aid schools to identify new ways to recruit candidates into their program. Thus, it is important to evaluate the factors that can influence a candidate's likelihood to pursue pharmacy school.

A number of studies discuss pharmacy program recruitment strategies and impact of different factors on prospective pharmacy students' choice.^[^12, 13., 14^]^ This study was different from prior studies assessing trends in pharmacy school admissions as it is the first study assessing the candidate pool using Reddit data as a survey.

The purpose of this study, which was conducted through an online survey, was to describe prospective candidates' perspectives and attitudes toward pharmacy as a career pathway. The study also examined appealing and unappealing characteristics about a career in pharmacy in potential pharmacy candidates and assessed factors that motivate or deter them from considering a specific pharmacy school. The study findings will provide insight to pharmacy programs as well as other stakeholders such as pharmacist employers regarding the perspectives of the next generation of students considering pharmacy as a profession. Hopefully by raising awareness of areas of concern, appropriate strategies can be utilized to address these barriers.

## 2 Methods

A cross-sectional study design was used to assess the perspectives and attitudes of prospective healthcare candidates toward pharmacy as a career pathway. A 28-item online Qualtrics open survey was disseminated through Reddit from October 4, 2022, through October 12, 2022 using convenience sampling. The inclusion criteria were any person considering pursuing a career related to health sciences and healthcare who had access to the social media platform Reddit. Exclusion criteria were anyone who did not intend on pursuing a degree or studying in health sciences and anyone who did not consent to the survey. The use of survey methodology allowed for gathering perspectives of a large number of prospective candidates during a specific time period; this snapshot aligned well with the study objectives.

Reddit was used for survey dissemination as it is a social media platform, consisting of hundreds of subcommunities known as subreddits, that allows for exchange of ideas and discussion regarding various topics. This social media platform is used by pre-health students to discuss career options. Two of the investigators searched for relevant healthcare and related subreddits. Search terms included pre-health, pharmacy, pharmacy school, healthcare, nursing, pre-nursing, physician assistant, and medical school. Once these words were entered in the search bar, the related subreddit suggestions would populate under. The investigators selected from possible options. They adhered to the subreddit posting rules. If a survey link could be shared to a subreddit according to the posting rules, the investigators reached out to the moderators of the group to obtain approval for posting. This process resulted in the study information and survey being posted to the r/nursing, r/samplesize, r/surveycircle, and r/takemysurvey subreddits. The completion of the survey was voluntary.

Researchers at Touro University California College of Pharmacy in Vallejo, California designed this study. The study was approved by expedited review by the conducting university's Institutional Review Board. Personally identifiable information was not collected. Only the investigators had access to the survey data. The survey included an informed consent and comprised two parts. Forward and backward navigation was possible, allowing respondents the option to review and change answers. The survey consisted of 8 pages and 26 questions total. The first part asked about participants' demographics and current level of education/degree. The second part assessed participants' overall perspective on pharmacy as a profession in the United States. Questions were developed by the investigators with reference to previous relevant work, specifically in some answer options such as areas of ranking important factors when selecting an educational program (proximity to current residence and proximity to family/ friends) as well as factors preventing participants from pursuing a degree in pharmacy (family obligations, having to borrow too much money in loans to pay for education, the pre-requisite courses).^[^^13^^]^ The literature analysis allowed for identifying relevant themes that informed the construction of the specific survey questions.

All statistical analyses were conducted using Stata IC 15 (College Station, Texas). Descriptive statistics were used to present the survey data. Continuous data, such as age, are described by means and standard deviations. Most of the data assessed were categorical in nature. This included additional demographics, important factors in selecting an educational program, factors preventing participants from pursuing a pharmacy degree, perceptions of the profession of pharmacy, and sources used to research professional school. Categorical data are reported in frequency and percentages. All survey responses that included more than just demographic information were included in the analysis. Surveys were excluded if they provided insufficient data or duplicate responses. The number of participants (N) for each question may vary with one another as not all participants answered all the questions in survey.

A sample size calculation was completed to ensure a 95 % confidence interval estimate with an estimated 5 % margin of error. A response distribution of 50 % was selected to estimate the largest sample size needed. A sample size of 385 participants were needed according to the sample size calculation. This means 385 or more measurements/surveys are needed to have a confidence level of 95 % that the real value is within ±5 % of the measured/surveyed value. As such, the study was designed to include enough participants to adequately address the research question.

This study was self-funded. Participants who completed the survey were given the opportunity to enroll into a raffle to win one of ten $10 Amazon gift cards. The gift cards were awarded randomly and sent through email to the participants as an incentive to motivate or encourage them to participate in the study. The study meets all ethical requirements as per Elsevier guidance. The relevant items from the Checklist for Reporting Results of Internet *E*-Surveys (CHERRIES) have been reported.

## 3 Result

A total of 526 participants completed the survey. Of these participants, 400 (76 %) were included in the study analysis. The rest were excluded due to insufficient data (54) and duplicate answers (72). The baseline characteristics of the participants are shown in Table 1. Overall, participants had a mean (SD) age of 25 (5.4) years and were predominantly male (66 %). Most of the participants identified as White/Caucasian (78 %), and most participants were from the United States (79 %). Of the participants from the United States, Californians had the highest number of participants 70 (17.6 %). Most of the participants (90 %) revealed that they had work or volunteer experience in a pharmacy setting. Additionally, 96 % of participants indicated they are planning on applying or already applied to a pharmacy school program. When asked about amount of research done to investigate pharmacy as a potential career path, 180 participants (45 %) revealed that they did a moderate amount of research, and only 13 participants (3 %) did a great deal of investigation about considering pharmacy as potential future career.

**Table 1:** Demographics of Prospective Healthcare Students Who Participated in the Survey.

Participants	M (SD)	Frequency (%)
Age (*N* = 383)	25.4 (5.4)	
Gender (*N* = 400)		
Male		265 (66)
Female		115 (28)
Trans female/Trans woman		5 (1)
Trans male/Trans man		5 (1)
Genderqueer or nonbinary gender		5 (1)
Preferred not to answer		5 (1)
Ethnicity (N = 400)		
White/Caucasian		312 (78)
Multiple races		32 (8)
Hispanic/Latino/Latinx		19 (5)
Asian/Pacific Islander		13 (3)
Black or African American		12 (3)
American Indian or Alaska Native		8 (2)
Prefer not to answer		4 (1)
Country (*N* = 352)		
USA		279 (79)
Others		73 (21)
State (*N* = 397)		
California		70 (18)
Alaska		27 (7)
Alabama		25 (6)
District of Columbia		25 (6)
New York		19 (5)
Florida		19 (5)
Colorado		19 (5)
Other states		193 (48)
Highest Level of Education (N = 400)		
Some college		153 (38.25)
Graduate degree		113 (28.25)
College degree		75 (18.75)
Post-graduate training		37 (9.25)
High school degree or equivalent		17 (4.25)
Preferred not to answer		3 (0.75)
Some high school		2 (0.5)
Type of Degree Currently Completing (*N* = 396)		
Bachelor's degree		153 (38.64)
Graduate degree		122 (30.81)
Associate degree		62 (15.66)
Post-Graduate training		46 (11.62)
High school degree		11 (2.78)
Other		2 (0.51)
Worked/Volunteered in Any Type of Pharmacy Setting (N = 400)		
Yes		359 (90)
No		32 (8)
Preferred not to answer		9 (2)
Amount of Research Done on Pharmacy as a Potential Career Path (N = 400) A moderate amount		180 (45)
A lot		104 (26)
A little/None		103 (26)
A great deal		13 (3)
Planning on Applying/Already Applied to a Pharmacy School Program (N = 400)		385 (96)

Table 2 shows factors ranked by the participants as the most important in choosing an educational program to pursue a degree in pharmacy. Of 400 participants, 119 (30.91 %) of them had the North American Pharmacist Licensure Examination (NAPLEX) pass rate as the first important factor and 94 (24.42 %) of them picked the number of graduates matched with Post Graduate Year 1 (PGY1) residencies or fellowships as their first important factor.

**Table 2:** Ranked Important Factors in Selecting an Educational Program to Pursue a Pharmacy Degree (*N* = 385).

	Ranked importance N(%)					
Answer options	First	Second	Third	Fourth	Fifth	Sixth
NAPLEX pass rate	119 (30.91)	53 (13.77)	11 (2.86)	1 (1.04)	1 (0.26)	0 (0.00)
Number of graduates matched with PGY1 residency or fellowship	94 (24.42)	89 (23.12)	49 (12.73)	16 (4.16)	4 (1.04)	14 (3.64)
Success of programs' graduates on obtaining jobs immediately after graduation	51 (13.25)	33 (8.57)	23 (5.97)	14 (3.64)	2 (0.52)	3 (0.78)
Proximity to current residence	50 (12.99)	50 (12.99)	34 (8.83)	8 (2.08)	2 (0.52)	0 (0.00)
Proximity to family/ friends	40 (10.39)	50 (12.99)	54 (14.03)	10 (2.60)	4 (1.04)	0 (0.00)
Reputation of the program	31 (8.05)	38 (9.87)	27 (7.01)	8 (2.08)	1 (0.26)	0 (0.00)

NAPLEX: North American Pharmacist Licensure Examination, PGY1: Postgraduate Year One.

When selecting the most important factor that impacted their decision to choose an educational program to pursue a pharmacy degree, the duration and reputation of the program were the most prevalent answers, and each have been picked by 22.34 % of participants as the answer to this question. Distance from current residence, cost of the program, location, recruitment, availability of online or hybrid option and the option “other” were selected as the most important factor by 15.84, 15.39, 12.99, 6.23, 4.42, 0.52 % of participants, respectively.

Table 3 demonstrates participants' choices when asked which factors are preventing them from pursuing a degree in pharmacy. Lack of opportunities after graduation stood out as one of the main concerns selected by 179 (44.75 %). A total of 145 (36.25 %) of people said having to borrow too much money (in loans) to pay for education is one of the factors preventing them from pursuing a degree in pharmacy.

**Table 3:** Factors Preventing Participants from Pursuing a Degree in Pharmacy (N = 400).

Answer options	Frequency (%)
Concern over the lack of opportunities after graduation	179 (44.75)
Having to borrow too much money (in loans) to pay for education	145 (36.25)
Family or personal obligations	123 (30.75)
Lack of letters of recommendation	114 (28.5)
Planning on pursuing other pathways after graduation	106 (26.5)
The pre-requisite courses	104 (26.00)
Lack of information to consider pharmacy as a career	58 (14.5)

Positive or negative things that participants have heard about pharmacy are included in Table 4. A total of 273 (68 %) participants heard that there are a variety of job opportunities in many different types of settings of pharmacy and 182 (46 %) of them heard that a career in pharmacy includes opportunity for direct patient interaction. Of the participants, 176 (44 %) had learned that pharmacists have a high salary, 211 (53 %) heard the pharmacy field has become heavily saturated, 174 (44 %) said that pharmacists appear overworked and pharmacy environments appear understaffed, and 156 (39 %) believed community pharmacists have a negative outlook on their jobs.

**Table 4:** What Participants Had Heard About the Profession of Pharmacy (*N* = 399).

Positives	Frequency (%)	Negatives	Frequency (%)
The variety of job opportunities in many different types of settings	273 (68)	The pharmacy field has become heavily saturated	211 (53)
Good opportunity for direct patient interaction	182 (46)	Pharmacists appear overworked and pharmacy environments appear understaffed	174 (44)
High Salary	176 (44)	Community pharmacists have a negative outlook on their jobs	156 (39)
Possibility of remote work (With telehealth – allows pharmacists to care for patients using a laptop computer or secure telehealth platform)	162 (41)	All they do is fill and checkoff prescriptions	152 (38)
Interaction with other healthcare workers	156 (39)	Possible liability from mistakes	137 (34)
Flexible scheduling	155 (39)	Pharmacists are underappreciated by the public	135 (34)
Establishment of status within healthcare	150 (38)	The cost of completing a pharmacy education is too expensive	124 (31)
You will have a stable career	133 (33)	Need for additional certification or residency to stand out among increasing competition	116 (29)
There is an increasing demand for pharmacists	128 (32)	It takes too long to complete a pharmacy degree	104 (26)
Available job benefits	124 (31)	There are limited promotion opportunities for pharmacists	89 (22)
You can run your own business by opening your own pharmacy	94 (24)	Other healthcare professionals have higher salaries	79 (20)
You will be helping patients to improve their health	72 (18)	Pharmacy jobs have high physical and mental demands	39 (10)
You can move anywhere in the United States and still be able to find a job	35 (9)		

Table 5 summarizes distribution of participants' selected choices in response to the question about the resource the prospective candidates typically use to research into pharmacy schools or other healthcare professions. Of the 399 participants who responded to this question, 238 (60 %) use social media platforms and online forums (Reddit, Instagram, Facebook, etc.), 193 (48 %) noted benefit from career day at their college or university, and 174 (44 %) use career counselors' help at college or university to research into pharmacy schools or other healthcare professions.

**Table 5:** Sources Typically Used to Research Pharmacy Schools or Other Healthcare Professions (N = 399).

Answer options	Frequency (%)
Social media platforms and online forums (Reddit, Instagram, Facebook, etc.)	238 (60)
Career day at my college or university	193 (48)
Career counselor at my college or university	174 (44)
Friends or family	153 (38)
College of pharmacy websites	148 (37)
Healthcare/recruitment fairs	128 (32)
Other healthcare professionals working in the field (nurses, physicians, and physician assistants)	102 (26)
Pharmacists	86 (22)

## 4 Discussion

Using survey methodology disseminated via the social media platform Reddit, this study assessed prospective candidates' perspectives and attitudes toward pharmacy as a career pathway, examined appealing and unappealing characteristics about a career in pharmacy in potential pharmacy candidates, and evaluated factors that motivate or deter them from pursuing pharmacy as a career. Many participants selected NAPLEX pass rate, PGY1 residencies or fellowships, and duration and reputation of the program as important factors in choosing an educational program to pursue a pharmacy degree. Most participants had concern over lack of opportunities after graduation and the field being heavily saturated while many of these participants heard that there are a variety of job opportunities in many different types of settings of pharmacy. Most participants typically use social media platforms to research into pharmacy schools.

This study differed in design and dissemination from previous studies assessing pharmacy school admission trends as well as motivators and deterrents to pursuing the field of pharmacy. For example, a former study in this area was done through a mail survey that was sent to students who intended to major in pharmacy at a large midwestem college of pharmacy.^[^^15^^]^ The use of an online social media platform allowed this study to be completed in a short period of time and for the study to reach a broad, diverse group of participants. Social media dissemination of surveys allows for ease of access for participants. The use of the social media platform Reddit, with the advantages of reaching a diverse survey participant pool efficiently, allowed for greater participation and generalizability of the results. Additionally, this study was different from prior studies assessing trends in pharmacy school admissions as it is the first study assessing the candidate pool using Reddit data as a survey. According to a survey conducted in 2021, Reddit is most popular among users aged between 18 and 29 years old and generally more popular with men.^[^^16^^,^^17^^]^ This is a relevant age group to target as majority of individuals who apply for a pharmacy program fall in the same age category.^[^^6^^]^ The broad geographical representation of participants enhances the generalizability of the study's findings. Another factor that differentiates this study is that the study survey was available for participants in the entire United States as well as other countries. This study had participants from all over the United States with representation from various parts of the country unlike other studies that focused on one location.^[^^15^^]^

An unexpected finding of the study was that though most participants had heard that there are a variety of job opportunities in many different types of settings of pharmacy, many also expressed concern over the lack of opportunities after graduation. Also, many were hesitant to pursue the field of pharmacy due to feeling that it is saturated. This may be due to prospective candidates having heard that a variety of job opportunities exist for pharmacists, but not really being aware of exactly what those opportunities are in the workplace. One suggested explanation for this can be that candidates are more aware of traditional roles of pharmacists and may not be fully aware of all the opportunities available to them post-graduation. However, today, the different areas of pharmacy practice are extensive and the field is continuously advancing to meet the needs of the patients being served.^[^^18^^]^ Roles available to pharmacy school graduates are by no means limited.^[^^20^^,^^21^^]^ In fact, in many states pharmacists have healthcare provider status and are authorized to provide health care services in diverse settings. New strategies are needed to provide information to potential pharmacy candidates on types of pharmacy work opportunities available postgraduation. This survey participant group indicated typically using social media platforms and online resources to research healthcare professions. As such, effective strategies to disseminate information regarding diverse career paths in pharmacy include educating prospective candidates about reputable websites on pharmacy career paths, pharmacy specialties, and post-graduate residency and fellowship opportunities.

Considering the study results, an interesting finding was that many participants had either NAPLEX pass rate, or the number of graduates matched with PGY1 residencies or fellowships as their first or second important factor in selecting an educational program to pursue a pharmacy degree. Likewise, we learned that duration and reputation of the program are among the two top important factors in selecting an educational program to pursue a pharmacy degree. These outcomes would assist pharmacy programs to understand candidates' viewpoint and accordingly help programs focus on candidates' area of priorities when selecting a pharmacy school. With these insights, pharmacy programs can target recruitment strategies, particularly online on program websites, toward highlighting and featuring NAPLEX pass rates, success rates of graduates in residency and fellowships, and information about their program.

Our study results show that most participants used social media platforms and online forums (Reddit, Instagram, Facebook, etc.) to research pharmacy schools or other healthcare professions. Schools and college applicants are increasing relying on social media platforms and online forums of prospective schools to obtain information for their enrollment decisions.^[^^22^^]^ According to a 2016 report, 50 % of students surveyed used social media to investigate about their potential future school, and 80 % considered and relied on the information gained from conversations with current students on social media when making their final decisions.^[^^22^^]^ The right choice of social media platforms, content, and audience can lead to more engagement and success in recruitment.^[^^22^^]^ For example, Facebook can be used when the goal is to strengthen brand and reputation. On the other hand, twitter may be more effective as a quick mean for interaction and sharing news.^[^^22^^]^ When connecting with working professional and alumni, LinkedIn may be the most effective channel. Social media currently plays an important role in prospective candidates' decisions about a program. As such, online presence of programs can play a beneficial role in recruitment and engagement of prospective candidates.

These study findings can be used in enhancing pharmacy programs' recruitment strategies. Some of the strategies can be easily implemented by pharmacy programs. For example, necessary changes and updating content on the school website as well as social media to reflect program-related statistics and other efforts to educate prospective candidates about the field of pharmacy, especially career options postgraduation, can be effective in providing accurate information about the field of pharmacy and can increase candidates' interest. Dissemination of specific program related statistics, such as those related to graduates' successful residency and fellowship placement, can allow for programs to stand out and showcase positive aspects of their school.

Though the study had multiple strengths, it also had several limitations. The findings may not be generalizable to all prospective pre-health students as this survey used Reddit for recruitment. Even within the Reddit platform, the investigators were limited to subreddits that allowed for posting survey links and approved the posts. The survey participants were predominantly men. This does not represent the majority gender distribution of current pharmacy students' population in the United States as majority of this population is comprised of women (59.6 %).^[^^23^^]^ However, the study result can provide valuable data about male candidates' perspectives and attitudes toward pharmacy as a career pathway and hint at strategies that may be effective in attracting this group to the profession. Additionally, trans female/trans woman, trans male/trans man, genderqueer or nonbinary gender, and people who preferred not to reveal their gender, comprise only 5 % of study participants. This proportion may or may not represent an actual ratio of this population in pharmacy and other health care programs as available data from population-based surveys may be limited due to under-reporting and imprecise results due to the existence of societal barriers. The potential lack of generalizability to all prospective pre-health students due to the gender distribution bias within the sample can be addressed in future research allowing for a more comprehensive understanding of diverse candidate perspectives. Another issue is that recall bias was a limitation. Using focus groups or open-ended survey questions can be helpful methods in addressing recall bias in turn leading to more accurate information about participants' perceptions regarding the field of pharmacy. This limitation can specifically affect the result of questions assessing what the participants have heard about the field of pharmacy. These types of questions rely directly on the participant's memory.

## 5 Conclusion

Although the majority of participants heard that there are a variety of job opportunities in the field of pharmacy, most of them still had concern over the pharmacy field being heavily saturated and lack of opportunities after graduation. Most responders gained this information through social media platforms. This emphasizes the importance of providing prospective candidates with relevant information and choosing the right means to do so. For prospective pharmacy school candidates, NAPLEX pass rate, number of graduates matched with PGY1 residencies and fellowships, and duration and reputation of the program seems to be among the most important factors in choosing a pharmacy program. These findings can be valuable and have practical significance for pharmacy programs, informing targeted recruitment strategies for attracting pharmacy students.

## CRediT authorship contribution statement

**Shadi Doroudgar:** Writing – review & editing, Writing – original draft, Visualization, Validation, Supervision, Project administration, Methodology, Investigation, Formal analysis, Data curation, Conceptualization. **Mahsa Sedaghat:** Writing – original draft, Visualization, Formal analysis. **Nabeez Noor:** Methodology, Investigation, Data curation, Conceptualization. **Shervin Gorji:** Methodology, Investigation, Data curation, Conceptualization.

## Declaration of competing interest

The authors declare that they have no known competing financial interests or personal relationships that could have appeared to influence the work reported in this paper.
